# Supplementary material for: Heterogeneous nuclear ribonucleoprotein E1 binds polycytosine DNA and monitors genome integrity
Source: Life Sci Alliance. 2021 Jul 16;4(9):e202000995. doi: 10.26508/lsa.202000995 (PMC8321654; doi:10.26508/lsa.202000995)
Supplement: Supplementary file 1 [file LSA-2020-00995_TableS1.docx]

**Table S1. NMuMG sequences of 50-75 nucleotides with at least 50% ‘C’, which includes sequences with ‘C’ clusters and potential hnRNP E1 binding sequences.** All ‘CCC’ repeats are highlighted and some potential hnRNP E1 binding sites are underlined. No line/fragment is contiguous with the preceding or succeeding sequence. We have identified over 30,000 fragments with at least 50% ‘C’. The first 368 fragments are shown here. The full list of 30,000+ fragments are available upon request and will be deposited on GitHub or another public access repository upon publication of the manuscript.

AACCTCCGCCGCCCTAGACCGATCCCGGGGCGCCGCCCCCTTCCCGGTCCCGCGCCTCACCTGTGCGCCCGCAAC

TACATCCAGCTCACTGCCCCCTCCCGGTTACCCCTCCCACAATCCTTTCCCCTTCCCTCCCCTTCTTCTCTGATT

CCTCTCCCTCTCTGTCTTCCTTTTCCTCTCCCACTCCAATAACCCTGCCCCCCCCCCCCCCCCCCCCCTTCCTCC

CCATGCAAGTACCTCCCCCCCCGCCCCCCCAACACACAAGGCCCAAACAATTCACCCCTCTAAATAACCCCCTCC

CTCCTCCCCCCTTCTCCTTCTCCTCTTCCTCCTCTTCCTCCTCCTCCTCCTCCTTCTTCCTGATCTTCCTCCTCT

GGCAAGCCCCACCACTGCCGCCGCCGCCACCCGGCTGCCAGGCCCCATCGCCCCCGCCCGCGGCGCCACCCTCAG

CCAGACTTTCCCACCAAACCCCAGTATCTCCCTCCTGAACTCCAGCATCTCCCACCAGACCCCAGCATCCCCCTC

CTCCTCCTCCTGCTCCTCCTCCTGCTCCTCCTCCTCCTCCTCCTGCTCCTGCTCCTCCTCCTGCTCCTGCTCCTC

CTCCCCTCCCCTCCCCTCCCCTTCCCTTCCCTTCCTTTCCTTTCCCTTCCCTTCCCTTTTCTCTTTTTTTTATTT

AAAATCACCTATCTCCTCCCCCTTCCTCCTGCTCCCTAACCCACCCACTCCCATTCCTGGTCCTGGCATTCCCCT

ATCCCCTTTCCACCTCTCTCTCTCTCTCTCTCTCTCTCTCTCTCTTCCTCCCTCTCTCTCTCTCTCTCCCCTTTT

AGGTCGAACACGTGGGTGTGCCACTCCCCCCCCCCCCACCCCCGACCTCCCCTCCCGCGCCGAGCCCGACCCCCC

TCCTAGTTTCCCCTACGAAATTCCTCTATCCCCTCCCCCTTCCCCCTGCTCCCCAACCCACCCACTCTTGCTTCC

CTACCGTTCCCCCAGCCCTGGCCCCCAAGCCCTGTACTCTAAGCTCTGTACCCCCAGTCCTGCCACCCAGCCCTG

ATAATCGACCATGATGCCCTCCCTCCAGCCCCCCCGGGCGCCCCGCCCCGTCACTTCATCACGGCCGCGCGCGCC

CTTCCCTTCCCTTCCCTTCCCTTCCCTTCCCTCCCCCTCCCCCCCCCCCCCCTTTCCTTCCTTTTTTTTTTTTTT

TACATTTCCAATGCTATCCCAAAAGTCCCCCATACCCCCCCCCCCCACTCCCCCCCCCACCCACCCCCCCCTTTT

CCAGCCTGCCCCGCGCGCGGCCTCTGCGCCTGCGCGCGCCCGGCCACCCGACCCCGAGGCGGCCCGGCCGGGAGG

CCCCGGCTTTGGGTGACCCCTGACCCTGGCCGCCTGGGCTCGCCTTCCCGCACATTCCGTCCTCGCCGCCCCCCC

TTTCCCCTCCGAAAACCCCCTATTTCCTACCCCTTCCCCTGTTCACCAACCCACCCACTCCTGCTTCCTGGCCCT

CACCCTTCCCCACCCCTTTCCCTTCTTAAAGGAATGCCATCTTCCCCTAATCACCTCCTTTCCTCTCACCCCACC

TCAGTTCCGCCTACCACGGTCCTCTCTCCCCGACAGCCCAACCACGCTAACCCCAGGCCCTTCGTCGCCCGCGTC

TGCCCTCCTCCTGGTCACCCCCCCCCACAAATATACCCCCCCCCCCCCCCCCTCCCCCCTCCGCCCAATAATACC

TCCCCTCTTCCTACCTTGTTCTCTCTCTTCCTCCCTCTCCCCTCTCTCTCAGTCCCCCCTTTCTCTCTCCCTTCT

ATTCACGGCAGCCTCCTCCAGGAGCGCCATCCCCCGCACATCCCGCCACACCGTCTCTCCGCCCCGCAGCACACC

CCATACACCCACCTATCCACCTGTCCATCCATCCATCCATCCATCCATCCATCCACCCACCCATCCATCTGTCCA

CTCTCTCTCCTTCCCCTCTCCCTCTTCCTCCCCCCCCCCCCCCCCCCCCCCCCCCTTCCTCCCCCCCCCTTCCTC

CTCTCTCTCTCTCTCTCTCTCTCTCTCTCTCTCTCTCTCTCTCCCTCTCTCTCTCTCTCTCTCTCTCTGTCTCTC

TTCTCCTCCATCTCCTCCATCCCCTACCCATCTCCTCCTCCATCTCCTCCCTCCCCTACCCATCCCCTCCTCCCC

GCACGCACGCACACGCGCAAACACACACAGCCAGACCCAGACACGCAGCACGACGCCCTCCCTCCTCCCTTCTCC

CCCTTTCCCCTGCTTCTATGAGGGTGTTTCCTCACCCACTCACCCACCCATCCACCCATTCCCACCTCCCTGCCT

CCCCTATCCTCCTTCTCCTCTTCTCACAGTCCTCCCCCTCTCTCTCCCTCCTCCCTCTCTCTCTCTTCCTCCTCC

CCCACCCAGCCGCGCGGCCCCCTCACGCCGCCGGGCGGCCCGATTTCCAGCAGCTTCCAGAGCCCCATCCCCACC

ACAGAACCCATCCCTCCTCCCTGCCCCCCCATAGAGTCCCTACCCTCATCTTCCCCCCCCTTCTCCTCTGAGAGG

CAGCTGCTTCCTCCCCCTCCCCCATACCTCTCACACGCACCCCTTCCCCCCTACACTGGAGCCTTAGATCCTAGC

CCTCTCTCTTCCTCTCTCCTCTCCCCCCCCCCCCCCCCCTTCCTCTTCCTCTTCCTCCCTCCCCCCCTTTCCCTT

CCTTCTCCTTCTCCTTCTCCTTCTCCTTCTCCTTCTCCTTCTCCTTCTCCTTCTCCTCCTCCTCCTCCTCCTCCT

CTATCTCATCCTCCCTCCCCCTGCTTCTATGAGGGTGCTCTCCCACCCAGCCTCCCACTCCTGCTTCTCCACCCT

TTTTCTTCTCTTTTTCCTCCTCCTCCTCCTCCTCCTCCTCCTCCCCCCCCCCCTCCCCCTTCTTTTTCCTTTTTT

CCTCCTCCTCCTCCTCCTCTCTCCTCCTTCTCCCTTCTCCTCCTCCTCCTGCTCCTCCTCCTCCTCTCTCCTCCT

TCCTTCTCCTTCTCCTTCTCCTTCTCCTTCTCCTTCTCCTTCTCCTTCTCCTTCTCCTTCTCCTTCTCCTTCTCC

CCCCTACCTACCCACCCACTCCCTCCTTGCCACCTTGCCATTCCCCAACACTGGGGCACTGAACCCACACACACC

CCTTCTTTCCTTCCTTCTTTCCTTATCTCCCTCCCTCCCCCCCTCTCTTCTTCCTTTCTCCCCCCCCCCCCCCCC

CTACAACAGTTTCCCCTCCCTTTGTTCTTCCAAGTGCTTTGCCCCCCCCCCCCCAACACTCCCCCCTCCACCCCC

CCTCTCCCCTTCTCTCCCTCCTCCTCTTCTCCTCCCCCTCTTCCCCCTCCTCTTCTTCTTCCTCTTCCTCCTCTT

CTTCCTCCCCTTCCTCCTCCTTCCTCCCCCTTCCTCCTCCTTCCTCCCTCTTCCTCCTTCCCTTCCCTCCCTTCC

TGTGCGCACACACTAGAAGTTTCCAGCCCCCCCCCCCCCCCCCCCTTCCCCCCCCCCCCGGGCACTCGGGGTTTT

CGCCCCCCCAAGCCCAGGCCGGCCCTAACCCACCCGCGGCGCGCTCCGGGACACCCCGCCGCCTCTCACGGCCCT

ACATTTCAAATGCTATTCCCAAAGCCCCCTATACCCTCCCCCGCCCTTCTTCCCAACCCCCCCACTCCTGCTTCC

TCCACAAAACCTCCACCCCATTTCCCTCCCCTTTGCCTCTAAGAGGGTGCTCCACCACCCACCCACTCCCACCTC

TCAGTCCATCCTCCCTCAACCTCAGCCCATCCTCCCTCAACCTCAGTCCATCCTCCCTTAACCTCAGTCCATCCT

GCTACCTGCTGAGAGCCCCCCCCCCCCCCCACCCCGCCTCCCCCCACCTCCCCCCCCCTCGGCCCAACCCCCCCC

CCTCCAGCCCACCACCACGTGACTCCGACCTCCAGCCCACCACCACGTGACTCCGACCTCCAGCCCACCACCACG

ACACCCCCCCTCTTGCTCTCACACCACAATCCGAGCCTGAGCCGAGACCATACCATCCCCACACGGCCCCCCTCC

CTCTCTCTCTCTCTCTCTCTCTCTCTCTCTCTCTCTCCCTCCCTCCCCCCTCCCTTCTCCTCACCCTCTGCCCAT

GTCATCCCCCTGCCTCAGAAAATCACTCACAGACACCCCCCCGCCCCAAAAAAACACTCACCACCCGCCCCCCCC

CCCTGCCCCTCCCGGTCCCTGCAGCCCACCTTGCTGCTCACCCCATCCTGTGCCATGCCCAGCCCTCCACCTCTC

CCTCTGCCTCTGCCTCTGCCTCTGCCTCTGCCTCTGCCTCTGCCTCTGCCTCTGCCTCTGCCTCTGCCTCTGCCT

ACCACCACAACCACAACCACCACCACAACCACAACCACCACCACAACCACAACCACCACCACAACCACAACCACC

TCACTCCTGATTCCCCACCACCCCAGTAACCTGATCCTTTTCCCCTTGGCCTCTCCCCCACCCCCGGCCCCCCCC

CTCATTTACATTTCCAATGCTATACCAAAAGTCCCCCATATCCACCCCCCCCCCCCCCCCCCCCCCCCCCCCCCC

CTACTGGCCCTGATCTAAGGACACAGTGTCAACTTCCCCACCCCCCGCCCCCCCCCCACCGCCCCCACCCCTGCC

GCACCCACAGCACCCACAGCAACCACTCCACCCATAGCACCCACTCCACCCATAGCACCCACTCCACCCACAGCA

GTTCCACATCCCATACCTCCTCCCACACCTGCCCCTCTCTCCACGTGGTTGTCCCCAACCCCCATCACACCCGAC

CCCAAAGTCCCCTATACCCCCCCCCCCCCCCGCCCCCTTCCCCCCCCCCCCCCCCTCTTTGGCCCTGGGGTTCCC

CCCTCTCTCCCTCCCTCTCTCTCCCTCTCTAATCTCCCTACCTAGTTGTCTCTTCCCCCCTTCAACTCCCCAACT

AGCAGCCAGTTGAGTGCTCCCTACCCTACCCCCACTGCTGCACAGTCCACCTCCTTCCCTTCTTCCCCCTCACCC

TTACCCCTCTTCTTCGCTCCGCCGCTCCGTGCGCGCCCCACTACGCTCCCCATTGACCCTGCGCTCGCCCCCTTC

CTCCGGAACCCCCCATCCCAATCTCCCCTCCCTGCTTCTATGAGGAGGCTCCCCCTCCCACCTACCCACACCCAC

GGCCACCCTCCAGCTCCCACCTCTGCTCTGGCTCCCACTCCCATGCCTGCCTCCACTACTTCAGCCTCAACTACA

CCAGGCGCCCCCACACCCACGCACCCTCCTGGCCCGGTACACCCCTCCCCCACGCCCGTGCGGGCGCACACCGAG

CTATATCCCTCCTCCTCCTCAAGCTCCACCCACTCACTACCACATCTATATCACTCCTCCCTCCTCAAGCTCCAC

ACTCTCCGGTGCGAAGGCCGCCCCTCCCCCTCCCCCGCCGCTTCCGCTCCGCGCCTCATGGGAGCCGGGCTCCTT

CCTCCCCCACCGCCCCACAAGAGTCTCCCACCACCTCCCATCCCAGGCTGGCCTCAAATTCACGATCCACCGCCT

ACAGCTGACCAGTGGACCCACAAGCCCCCCCTTCCCCCTGCGCACCCAGCTCTCGGGCCCACCCTGCTGTCTCAC

TCTCTTTGCACATCTGATCCCAACCCCACCATTCCCCTTTCCCCCTCCTCTTCCCCCCCATTCCCCCCCCCCATC

CCTCCTTTATCTCATGTCTTAGTCTCATCCTGGAGATGCACCCCCCCCCCCACCCCCCCACCCCCCCCCCCCCCC

ACACACATACCACACACACACCACACACACACACCACACACACACACACACACACACACACACACACACACACAC

CCCCAAATCCCCCTATACCCTCCCCCTCCCTGTTACCCCACCCACCCCCCCCTGCTTCCTTACCCCGGCATTCCC

CCACCTGCCTCTGCCTCCTGAGTTCCAGGATTAAAGGCATGCATCCTCCACCCCCCCCCCCCCCCTACCCCCCGC

TTTCCCCTCTGAAAGCCCTCCTATCCCATCCCCCCCCCTCCTGCTCACTAATCACCTATTCCTGCTTCCCTGTCC

GGCTCCGACCGAAGCCACCCGGGGCGGCCCCGCCGACCCCGCACCCGCCCCCGAGGCCCACCGACTCTAGTCGAG

CCGCTCCCCTTCTCCTCTGAGTGTGTGGAACCCCCCTCAGGCTTCGCACCCCCCCCCCGGCGCCCCCCCCCCCCC

CCCCTCCTCCTCTGGCTGCTCCGCGCTTCCTGCTGCCCACCCCACCCCCTCCCCGCGGGCTGAGTCCTGGCGTTC

CTCTCTCTCTCTCTCTCTCTCTCTCTCTCTCTCTCTCACACACACACACACACACACACACACACCCCTCTCTCT

CTCTCTCTCTCTCTCTCTCTCTCTCTCTCTCTCTCTCTCACACACACACACACACACACACACACACACACACAC

TTCTCCTCATTCCCCTCTTCCTCCTCTTTTCTTCCTCCTCCTCTTACCCCTCCTATCCCTCCTCCTCCTCCTCCT

ATCCACCCTTTCACCTCCACTCCCCAGCCCCAGCCCCAGCCCATACTCCTGCCAGGCCTCCCCACTCCCTGGGGC

ATACAGGCATTCCCCACCATGCTTCTTTTCCCCTTCCCCTTCCCTCCCGTTTTCCCTCTCTCCTCTCTCCCCTCT

TTCCCTTTCTCTCCCTTTCCCTTTCCCTTTCCCTCTCCCTCCCTCTCTCTCTCTCTCTCTCTCTCTCTCTCTCTC

ACATGTGTTCTCTCTCTCTCCCTCTCCCTCTCCCTCTCTCCCTCTCTCCCCACCACGCACATGATCACACACCAG

GTCCTGCCCCTTGTTTCCACAAGGATGGCCCCCCCCCCCCCCCCCCCACCCCCCAAATTTCCCAGGGACCTCCGG

CCCCTCCCAGCCCTCATTCATGAAGCCCTTTCTACCCAGCCCCACCCCTCCTGCCCGTTTCTTCTCCTCTGCCCT

TCTCTCTCTCTCTCTCTCTCTCTCTCTCCCTCCCTCCCCCTCTCCCTCTCCCTCTCTCTCTTTCCTGCTTACCTT

CCCACATGCTCCCCTACCCACTCCCCCACCCACCTACTCCCACTTCTTGGCCCTGGCGTTCCCCTGTACTGAGGC

CGCACGCACCCCCCCCCCCCCCCCCCCCCCTTTTCACACGCATTCCCTTTAAGAGAAATTTGAAACCCTGGCCGC

ACTCTATTGCCCTTGAAGCCCACCAAGAGCACCCTCCCAGAGCTGCCCACCCCCCCCCTCCCCCCCCCCCCAACC

CCCCTCTCCTAGTTTCCCCTCCCCAACCCCTTATCCCTTCCCCCTACCCCTGCTCACCAGCCTATCCACTCCCGC

CCAGTCACCATGGCGTCCAGCCTGTAAACCTGGCCCTCCACTGCCTCTCTCTCCACTCTACCCAGCCCCACCCCG

CCTTTCATGGTTTACCCTCCAAAGACCACCTATCCCTTCCTCCCTCCCCCTGCTCAGCAACCCACCCACTCCTGC

ATGCTGCGGCACACCCACCCCCGCCCCGCCCGCGAGTCCAGATCCGCCCCCCCCGCCCTTCTCTTCGACTGCATA

TCTCTCTCTCTCTCTCTCTCCCTCCGTCCCTCTCTCTCTCTCTCTCTCTCTCTCTCTCTCTCTCTCTCTCTCTCT

TCCTGACCCTCCCTCCTGAGCCCTCCTTCTCCTGAGCCCTCCCTCTCCTCCTTAGCTCCTCCCTCTCCTCCTGAC

TCCAATGCTATACCAAAAGTCCCCCATATCCACCCCCCCCCCCCCCCCCTCCCCCCCCACCCCCCCTTTTTGTCC

CTTCTCCTCCACCTCCTCCTCCTCCTCCTCCTCGTCCTTCTTCTTCTTCTTCTTTCTTCACCTTCTCCTCCTCCT

CCTTCCTTCCTTCCTTCCTTCCTTCCTTCCTTCCTTCCTTCCCTCCTTCCTCCCTTCTCCTCCTTATTCCTCCTC

TCCCATCTCCCCTCTTCCCTCTCCCCTCCGCCTCTATGAGGGTGCCCCTCCACCCACTCACCCACTCCTGCCTCA

TAACGGGGGCGCGCCGATACGGTCCGGCCCACCAGCCCCCCACCCCCCCCTCCCCCCCCCCACCCTCTCCCCGCC

CCCCCAGCATCCCTCCTTCCAGTTAAGCACAGCCTGTCTCCTGGCTCCTCCCGCCCCCCTTCTCTCGGGCCCTTT

TCTCTCTCTCTCTCCCTTCCTTTCTTCCTTCCTCCCTGCCTCCTTTCCTTCCTTCCTTCCTTCCTTCCTTCCTTC

TCTGTCCTCTCTTCCCGCTCTCCCTCTCTCCCCATTCCCCACCACCACCACCACATGCTCATGGCTGGCCTCTAC

CTTTTTCCTCTTCCCCTCCCCCTTAGTTACCCCCTGTTACCTCCTTCCCCCCCCCCCCTCTTTCCCCCCTCTTCC

CACCGCCTTCAGCCGGAGGCCGGAGTCTACCCTTCTCTCCGCCTCCCAGGGCCCCGCCCTTCTCCGGGCCTGCCC

TTCCCCCTTTCCTTTCCTTCCTCTCTCCCTTCCTTCCTTCCTTCCTTCCTTTCTTCCTTCCTTCCTTCCTTCCCT

CATAATGTCTTACAGCACCCCCCCCCCCCCCCCCCCCCCCCCCCCACCCCCCCCCACCACCCCCACACCGCGCAG

ACAGTACGCTCCCCGAGGAACCACAGCCCGGAGATCGCCCCGTGCCCCACCCACCCGCCCCGCGCACACAGGGCC

CCACCTCTTGGCTCCCTACATGCCAACCCCTCCGCATCTCTCCTCCTTCATCTCCACCCTCCTGCCCTGCATCCC

TCTCCTTCTTCTCCTTCTTTTCCTTCTTCTCCTTCTTCTCCTCCTCCTTCTCCTCCTCCTCCGCTTCCTCCTCCT

CCCATGCTACCAGTCACACCCACCCACACCTACCCCCCCCTACTGGGCACACCCCCCCCCACCTTCCCCCACCCT

GGCGTGCTCCACCACCGCCTGGCTGGTTTTGCTTTTCTTAAACAAGCCCCCCCCCCCCCCCCCCCCCCCTCTTCT

CTCTGAGACCCCCCCCCCCCCCAAAAACCCCCCGCCCCAACAAGCCCCCCCCCCCCCGGGGGGGGCCCCCCCCCT

TGTCTCTCTCTCTCTCTCTCTCTCTCTCTCTCTCTCTCTCTCTCTCACACACACACACACACACACACCCCCCTC

CCCTTTCCTGGTTTCCCCTCCAAAACTCCCCTATCCCCTCCCCCCCCCCCCCCCCCCAACCCACCCCCCTCCCCT

CCCGCCTGGCTCCCGCGCCCGCCGCGGGCCCCTACCAGGCCCGCCGCTCCGGGTCCCGCCCGGCTGGTTGCGGGG

CTCTTACACTCCCTCTTACACTCCCTCTTACACTCCCTCTTACACTCCCTCTTACACTCCCTCTTACACTCCCTC

CTCTCTCTCTCTCTCTCTCTCTCTCCCTCTCCCTCTTCCTCCTCCTCTCCCTCTCCCTCTTCCTCTCCCTCTTCC

AAGGAGCACACCCAGAAGCCAGAGCCAGGTCTCCTCCCCCCCCCCGCCCCCCCCCCGCCCCCCCCCCCCCCTCCC

CCTCCCTGCCCCCTTTCCCACCCAGGACGCTCCCTCCTTCCCCCTCCTGTGATTGCTTTCTCCTCCCTCCCAACT

GCACCACTCACTCACCCCCCCAGCCACACCACACACACACACACACACACACACACACACACACACACCTTTCCC

CTGTCGCCTCCGGCAACCCTGCGCTCCCACGCTCGCACCCCCAGTTCCCTGCAGCTCCCTGGCCCGCCCCTTGCC

TCTCTCTCTCTCTCTCTCTCTCTCTCCCTCTCTCTCTCTCTCCACCACCACCCTGCCCCTCTCTTTCTCTATAAC

TCCCCTTTCCTATTTCCTCCCGAAAACCCCCTATCCCTTCCCCGCTCTCCCTGCCTACCAACCCACACACTCCCA

CCCTAACCCTAACCCTAACCCTAACCCTAACCCTAACCCTAACCCTAACCCTAACCCTAACCCTAACCCTAACCC

CCTGCCCTTCACCTCTTGATCCCCTCGGCCTGTTACCTCCACCTTCAACCCTCATTACTGCTGCCCCACCCCCAG

TGCTCCTCCTCCTACTCCTCCTGCTCCTCCTCCTGCTCCTCCTCCTCCTCCTCCTGCTCCACCTCCTGCTCCTGC

GGCCTCGCCGCTGAGCGCCGCCCCACTCCCTTCCTCCCTCCCTCTTTACCTCCCTCCTCTCTTCCACTGCCTCCT

CTCTCCTCTCTGTCTTCCCCTCTCCTCTCTCACTCTCTTGCTCTCCTCTCACTCTCTTCCTTCTCTCCCTCTTAC

ATGCCACCTCCTCGCACCTCTCCACTGCGATTCCGCCTTGCTTCCCCCAGCCCACCCATCCGCCCCTAGGTGTCC

CAATACCAGATCAGGTTCCCCTCTACCACCCCCCCCCCCCCTCCCCCCCCCCCCCCTTTCCCCCCCCAGGACCCC

ACCCTATTCCCTCCCTGAATCCCATCTCCCCTCCCCTTTCCCACCTGACCCTCCCAGTCTCATTCTCCTGCCCTG

CCCAGCCCGAGAGGTCCTAGCCCTAGCCCCAGCCCCAGCCCTAGCCGCACTAGCCCTGGCCCCAACCATAGCCGC

CAGCCTGGCTCCCGCCCCCTAGGTGCCCACAGCCTGGCTCCAGCCCGGGTACCCACAGCCTGGCTCCCAACCCCT

CCCTCTCTCTCTCTCTCTCTCTCTCTCTCTCTCTCTCTCCCTCTCCCTCTCTCTCTCTCTCCCCTCTTCTTCTTT

CTCTTTCCTTCCTTCCTTCCTTCCTTCCTTCCTTCCTTCCTTCCTTCCTTCTCCTCCTCCTCCTCCTCCTCCTCC

CCCTCGCTCCCCACACCCTCCCTCCATCTCTGCCTTATTCTCTCCCCTTGTTCCCTCTTTCCCCTCCCTTCTTTC

CTCCTCTCCTTCCTCCTCCTCTCCCTCCTCCTCCTCTTCTTTCTCTTCCTCCTCCTCCTCCTCCCCCTCCTCCTC

CCCCTCTGCCCCCCCCCCCCCCCCCCTCCTCCCCCCTCCCCCCCCCCCCCCCCCCCCCCCCCCCCCCCCCCCCCC

CCAAGGCAGCTTCCATCTGCTTTCTCCTCAAACTCCCCCCCCCCCCCCCCCCCACCCCAACCCCTACCTCTCACA

CTCCTCCACCCCTCCTCATCTCCTCCTCCTCCACCCCTCCTCATCTCCTCCTCCTCCTCCCCCCCCCCTCCCTTC

TTTTTTTTCTTCTTTTTACAAACACTTGACCTCACCCCCCCCCCCCCCCCCCCCCCCCCCCCAACCTCCCTGCTA

ACTGCTCACCGTCCCGGCCCCCCCCCCCCCCCCCCCCCCCCCCCCCACCCACCCAACCAACCCCCCCCCCCCCCC

CTCAGCGCCCACTCTCCAGCCTCTGCCTCTGCTTGCAGCTCCCTCACTCCTCCCACCGTCCTGGACCTGCAATCC

CCAGACCCTTCCCCCCCCCCCCCCCCCCCCTCCTTGGCCCTGGGATTCCCCCCCCTGGGGCAAAAAAAATTTGCA

CACCCCTCAAACCGGCCGGGGACGCCACCCCCTCTCCCCCCGCCTCCTCGGCCTCCCCCACTCCCTGGCGGCGCG

CCTCCTCCTCTGCATCCTCTCCCTCTCCCATTTTCTCCTTCTTCTCTCCCCCAACTTTCAGCTCCACCTTCCCTT

CATCCTAGATCCCTGTGCCAACAACAGAGTGCCAAGTGCACAAGCACCCCCCCCCCCCCCCCACACCACCACCCC

CCCTTCTCCCTCTCCCTCCTCCTCCTCCTCCTTCTTCCTCCTCCTCTTCTTCTTCCCCTTCTTCTTCCCCTTCCC

TCGTCTTCCCTTATCCCCCCCTTCTTCCCCGCCCCTCACACTCCTCCTCCTGCGCACCCCGCTTTCCCTTGATAC

TCCCCGCAGCCGAGCGCACGTCCGCCAGCGCGGCCGGCCTCTGCTGGCCGCCGGAGCACCCCCCGCCGGGCCGCG

CCCTAAGATGAGGAACTGCTCTGAGAGAAATCCCCCCCGGCCCCCTCCCCCCCCCCCCCCCCCCCCCCCCCCCCC

CCTTCTCCTTCTCCTTCTCCTTCTCCTTCTCCTTCTCCTTCTCCTTCTCCTTCTCCTTCTCCTTCCCCTTCTCCT

TCACCAATTCCCATCCCATGATATCCCACTTCCAGCTTACCCCTCCAACAACCCCCAATCCCACATCTGCCTCCC

CACACACCCCTGCCATTCTGCACACTCTCACACCCCTGCCATTCTGCACACTCACACACCCCTGCCATTCTGCAC

CTTCCTTCCTTCCTTCCTTCCTTCCTTCCTTCCTTCCTTCCTTCCTTCCCTCCTTCCTTCCTTCCTCTTTTCCTC

CCCCGCCCTGCTTTCCCTCAGAAACCCCAACTCCAGATCCCCTCCTAGGCTCAGCAACCCCTACCCCCATCTGTA

TCCACCTTCCACATCCCACACATCCTCCCCACCCTGTCTCCACATGGATGACCCCACCCTCCACACTGCCTGACC

CTCTCTCTCTCTCTCTCTCTCTCTCTCTCTCTCTCCCTCTCTCCCTCTCTCTCTCTCTCCCTCTCCCTCTCTCCC

CACAGTCAGCCCCCAGTACACCTAGGCCCCAGTTCCCACCTCACAGTCAGCCCCCACCACACCCCGGCCCCAGCT

CCTCTGCCTCTGCCTCTGCCTCTGCCTCTGCCTCTGCCTCTGCCTCTGCCTCTGCCTCTGCCTCTGCCTCTGCCT

TATTTTTCTCACATATAGTACAGTCTGTCCCTTTCCTTCCCCCCACCCTCTCCCCCCCCCCCCCCCCTTCCCCCA

CCTTTCTTCCTCCTCCTCCTCCCTCCCCCTCCTCCCTCTCCTTCTCCAGCCCTTTCTCTCCCCTTGCCTTTCTCC

CTCCAAACCCGGTCATCTGACTCTCGCTGCCAGCTCCCCCGCCCCTACCACCCGTCTCCCAACCCTTGCAGCCTC

CTCCTACCTCTCCTCTTTCCCCTCCGCCTTCCCCTCCTGCTCCAGCTCCAGGTCCTGCTCCTGCTTCTCCTATCC

ATTTCCAATGCTACCCCATAAGTCCCCCCAACCCCCCCCCCCCCCCCCCCCCCCCCCCCCCCCCCCCCCAACCCC

ACCACAAACCCACGCTCTACCCACCCAGATCCCGCGGAGCGCATAGCACCCCTGGCCCCTAACTGCGCCCCGTCC

CCCCGAGGTCCCACCCTCCCCAAGGTCTCACCCTCCTTGAGCTCCCATCCTCCCCGAGGTCCCCTCCTCCCCTCC

CTCTCTCTCTCTCTCTCTCTCTCTCTCTCTCTCTCTCTCTCTCTCTCTCCCTCCTCCCCCCTTTTTTCCCCCCCC

GCCTGCTCCCGGTCGCCCCCTCCCGCGGCCCCGCGCCCCGCTGCCAACCGCCCCCGGCCGCACGGAGCTTTCTTA

GCCTTTGACATTTCTGAAGCCCACACCAGGCCCAGTCATAGCTCTCCCGCTCCCTCCCCCTCTTCCCCCCCCACC

CGACGACGTCCCCCGGGCCGTACGCACCCTCGCCGCCGCGTTCGCCGACTACCCCGCCACGCGCCACACCGTCGA

CTCACTCCCCTTCACAGTGTCACCCCTCCTCACTCCCCTTCACAGTGTCACCCCTCCTCACTCCCCTTCACAGTG

CCCTCCTCTTCTGCTCTACTCATCTTCTCTTCCCATCTGCTCCCTCCTCCTCCTCTTTCTCCTCTTCCTCCTCCT

CCCCATCTGCACCATCATGTGAGCCTTCTCTGTTACCCTAAATCCTCAGTTTCCCCCCCCCCCCCCACCACCCCC

TGCCCCTGTCTCTGCTGCCCCGGTTGCTGCCCCTGCCTCTGCTGCCCCGGTTGCTGCCCCCGCCTCTGCCACCCC

AAGCCCCCTACCAGCTTCCCCCCCTTTTTCTTTTTTTCCTCTCTCACCCCCCTCTCCCACCCCCATCTATGATTC

AACATTCCCTCCCTCCATTCCATTCTTTCTCCCTCTCTCCTCCTCTCCCTCCCTTCCTTCCTTCCTCTTCCTTCT

CCGCCATCCTTCCCCATCTCCCTGCCTCTCTCCCTCCTTCTGTCCCTCTGTCCCTCCCTCCTTCTGTCCCTCCGT

ACATTTACAAGGCTATCCCCCCCCCCCCTCCCCCTCCCTCCCCCTCCCCCTTTTTGGGCCCGGCCATTCCCCGTT

TCTTCTTCTTCTCCTTCTTCTCCTCCTCCTCCTCCTCCTCCTCCTCCTCCTCCTCCTTCTTCTTCTTCTTCCTCT

TTATCCCAAAAGTCCCCCATTCCCCCCCCCCCCCCCCCTTCCCTACCCCCCCCCCCCTCTTTTTTTGCCCCGGTG

CCTCTCTGCCTCTCTGCCTCTCTGCCTCTCTGCCTCTCTGCCTCTCTGCCTCTCTGCCTCTCTGCCTCTCTGCCT

ATCCACCACCACCAAAACTTTCTTTACTCCTTCCTCCCTCTCTCCTCTCCCTCTCTCCCTACTCGTCCTCCTCCT

TCCTCCTCCTCCTCCTCCTCCTCCTTCTTCTTCTTCTTCTTCCTTCTCCTTCTCCTTCTCCCTTCTTTCTCCCTT

CCTACCCATCTCCCCTTCCCCCTTCCCCCTGCTCACCAACACACCCACTTCCTGGCCCTGGCAGTGCCCCATACT

CGGTTCACCCAGCTGCACTGAGGACTTCCTCCTCCCCCCCCCCAGCCCCACCTCCCCTACCCCCCCCCCCCCCCC

CCCCTTGAAGTCCCCCCATCCCATCCCCCCATCCCTGTATCTCTGCCAGCCTCCATTGGGGACCTTCTCCCCCCG

TCCTCAAATCCTCTTAACCACTCCCCCCTCGCCCATCAACCCACCCACTCATGCTTCCTGGCCCTGGCATTTCCC

TGCTATCCCCTTTCCTAGTTTCCCTTCCAAAAATCCCCTATCCCCTCCTCCTCCCCCTGCTTCCCAACCCACACA

TGGCTCGATGCTCCGCTCAGTTCTCCTCCGCTTACTCCTCTTTTACTCACTCCCTTCCTCCCCCCCCATACCCCC

ATCCACCCCCCTTCCACCTCCATTCTTCCCCGTCCTCGCCGCCTCCCTTCGCCAATTTCGCCGGCCTAACCACAA

CGCCCCCATCTACTCTCTTACTGCTCCACATCCCACACCACTTCCCCACCAACCCTGTCTCCACCTGGATGCCCC

CCCCTTTCACTCACACACACACACACACACACTCACACACACACTCACACTCACACACACACCACACTCACACAC

CACACACACACACACACACACACACCACACACACACACACACCACACACACCCCACACACACACACACACACACC

CCTCCTCCTCCTCCTCCTCCCCCCCCCCCCCCCCCCTCTCCCTTCCCCCCCCCCCCTCCCTCCTTTTTTTTTTTT

CCCCCTTTCCTGGTTTCCCCTACGAAAACCCCCTATCTCCTTCCTCTCCCCCTGCTCACCAACCCACCCACTTCT

GTATTTATTTACATCCCAAATACTGCCCCCTGGGCCCCCTTCCCAGAGTTCCTACCCCCATCCCCCCCCCCCCCC

AGGAAAGATCTCCCTCCCTCCCTCCCTCCCTCTCTCCCCCTCCCCCTCCCTCCCTCCCTTTTTACCCATTTTCAT

CCCTCTCCACCTACTTCCCAATCTCTCTTCCCTTCATCCTCCTCATGGTCCTCTTCTCTCTCCTCCCTCCTCCTA

GAAAGTTCCCTATACCCTCCCACCACCCTGCTCCCCTACCCACCCACTCCCAATTCTTGGCCCTGGCATTCCCCT

CTGCTTTGCGCGCAGCTCCAAGCCCTTCTGCCCTTCCCCCACCCCAGCCTCCGTTTCTACCCTTCTTCCCGTCCC

CCCGCGCTTTCCCTGCCCGCCGCTCCGGATCGGCCCCCACCCGGGCTGTCCAGCGCCGCGGCGCCGCCAGGGACA

GCTTTTACCCCTTTCCCCTTGTCCCCTTTCCCCTTTCCACCTTTCCCCCAGCTCCCTTGCCCCCTTTTACCTTTC

CTGCGGCCGGCGCCCTCCTCCCCCCTTCTCCCCTCCTCCAGCCTGGCCTCACTTTCTCCCTGGGCTGGGCGCTTC

CCATAGCTGTGACCAGCCTGGACTCCTCACTGCCCCCCCCCCACACACACACACATACCCAGCCCCCCAAAAGCA

CTCCAAAATCCCCTATCCCATCCCCCTTGTCTCTATGATGGTGGTTCCCCCCCCCCCCCCCCCCCCCCCCCCCCC

GCTCTCCTGTTCTCTTACTCTCTTGTGCCTTGCTCCTGTGCTCCCCCTCTCTCCCCAGCCCCAGCCCCTTCCCGC

CCTCTTCTTCTTCCTCCTCTTCTTCCTTCTCTTCCTCCTCCTCCTCTTCCTCTTCCTCCTCTTCCTCCTTCTCCC

CTCCCCCTCCCCCTCCCTTCTTCCCCCCCCCTCCTTCCTTCCCTACAGTTGTTTAGGCCCTGCTATGACCCCTTT

CAGTCATCCCCGCCACTCCCCTGCAGTCATCCCCCCCCACCCCCCCGCCGCCATCCCCCCCACCCCCCCTCAGGC

GTTCTCGCCCATCCTTGTCGCCTCCTTGCCGCTCCCCTGGGTTTCCCCTCCCCACCTCTTTCCCTCTGACTCCCA

GCGTCAGCCGGATCCCGCGCGCCGCGGCGCAGCCCGCCCTCCTCGAGCTGGCTCCGCCCCACGCCTCCGCCCTCC

CAAGGACTCCTAAAACATGGTCACGCGACCCCCCGCCCCATCGCAGCACACCCCTCCACCACCCGTCATCACCTC

AGCTCATTTACATTTCCAATGCTATACCAAAAGTCCCCCTTATCCACCCACCCCCCCCCCCCCGCCCACCCCCCC

GCCCCCTATCCGCTCCCTCTTCCCCCTGCTCACTGACCCACCCACTCCTGCTTCCCTGTCCTGGCATTCCCCTAC

TTCCTCTCTGAAAATCCCCTATCCCCTCCCCTCTCTCCCTGCTCCCAAACCCACCCACTCCCCTTCTTGACCCTG

CCCTCTTTCTTCCTTCCTTTCTTCCTTCCTTTCTCTTTCTCTCTCTCTCTCTCTCTCTCTCTCCCCCTCTCTCCC

TCCCTTTTGGCCACGGCCACGGCAACCCGGCGACCCAGGCTCAGGCCAGCGCCATCCACACCCCACAACCCCCCA

CCACAGGCACCCCTTAAAGCAATTACCATCTATCCGCTTCCTCCACCCCTCTCCCCTCACCCCAGCCCGGCCTGA

TCCTTTCTTCCTTCCCTCTCTTCCCCCTTCCCTCCTTCCCTCTCTCCTTCCTTCCTTTCTTCCTTCCCTCTTTTC

TCCCCCGCGCCCCCGGCCACCGCCGTCTCGCGGGGCGCGTGCCCCCCCCGCCCCCGCGCCGTGTCCCCTCCACTG

TCCCCCCTTTCCTCTTTCTCCCCTTTTCCCCCTTTCCCCCCCTCCCCCCTCCCCCCCTCCCCTCTCCCTCCCCCC

GCCCCCTTAGCCTCCGGTCCGGCGGCGGCCCGGGACCCGCTCCCCGCCGGGCACTGACGCCGCCGCCGCCGCCGC

CCCGGCCCGAGCCCCACCCACACGACACGCCCCAGCCCACCGTGCCTCCTCGCTTCTTCGCGCTGCCTGCCTCAC

CCCATTCCCCTTCCCCTCTCTCCACGTGTTCATGGTTGACCTCTACTCCTCTACTCCCCCCTCCCCGCCTTTCTC

TCTTTCTCCTCCTCCTCTTCCTCCTCTTTCTCCTCCTCCTCCTCCTCCTCCTCCCCCTCCTCCTCTCCCTCCTCC

CCCCTTTCCCCTTTCTCTCTGTCTGGATCTCTCTCTCCTCCCTCCACCACGCCCCCTCCCCATCCTTTCTCTTTT

ACGCACCCTCGCCGCCGCGTTCGCCGACTACCCCGCCACGCGCCACACCGTCGACCCGGACCGCCACATCGAGCG

TGCTACATCCCTCTCCCCCCCCCCCCCCCCCCCCCCCCCCCCCCCCTTTCAAACCCGGGGCCGGGGGAGGAAAAG

CTCTATCCTGCTGCTTCTCCTGGCTGCTTCCCCCGCCCCCCTCCGCCACCGCCACCTTCCAGTCCACCATTTCCC

ACATGCGGCCGACACGGTGCCCTCCCCCCGTTCCCTCTGCACCTCCCCTGCCCCTGCACCCGCTGCCATCGCTCC

CCTCCTCTCCCTCCTCTCCCTCCTCTTCCTCCTCTTCCTCCTCTTCCTCCTCTTCCTCCTCTTCCTCCTCTTCCT

CGGAACTGGATTAGGCGTGTCCTCTCCCTCCGCCCCCCCCCCGCCCCTTTTCCCTCGGTTCACCCCCTCCCTCCC

TCTCTCCCATCCCTTCCAGTACCTGTTACTGCCTCCCTTATTTCCACCCCTCCTCTCTCCCTCCCAGGTCCGCCT

CCGCCATCCCATCCCTCCTCTCCTCCTCTCCCCTTTGGCTCTATGAGGGTGTTCCTCCCCCCACTCACCCACTCT

TATCCCAAAAGTCCCCATGCCCTCCCCTGCCACTTCCCTCTCCCCACCCACTCCCACTTCTTGGCCCTGGCGTTA

TATCCCAAAGGTCCCCCATATCCACCCCCCAACCCCCTACCCACCCACTCCCCCTTTTTTGGCCTGGCATTCCCC

TCTCTCTCTCTTCTCCCTCCCCCTCTCTCTCCTTCTCTCTCTCTCCTCTCTCTCTCTCCTTCTCTCTCTCTCCCC

CTTTATTCACATTTCAAATGCTACCCCGAAAGTTCCCTGTAACCTCCCCCCCCCCCCCCCCCCCCCCCCCCCCCC

CCTCTCCCAGTACCCCCTCCCACAGTTCCTCATCCCATTCCTCCTCCTCCCTGTCTCCAAGAGGAAGTCACTCCC

CCACTCCTGATCATGCTCCTCTTTTTCCCTCCTCCACCTTTCTCCTTCCCAGGTCCCTCCCTCCCTCTGCTTCCT

CCCACTGCACCTGTGCTTTTCCCTAGACTCTCCTTGCAGCCCCCCCCATTGCCACCCCACCCCCCCCCCCCCGCA

CTTCTCCTCTTCTCCTCTTCTCCTCTTCTCCTCTTCTCCCCCTCTCCCCTTTTCCCTCTCTCCTCTTTCTTCTTT

CCTTCCTTCCTTCCTTCCTTCCTTCCTTCCTTCCTTCCTTCCTTCCTCCCTCCCTTCCTCCTCTCTCCCTCTCTC

ACTTTACATCCCAATACCAGCCCCTCCTCTCCTCCCAGTACCTCTCACGCAGATACTCCCCCCATTCCACCCTCC

ATTTTCTTCATTTACATTTCCAATGCTATCCCAAAAGTCCCCCATGCCCCCCCCCCCCCCCCCCCCCCCCCCCCC

TTCCTTCCTTCCTTCCTTCCTTCCTTCCTTCCTTCCTTCCTTCCTCCCTCTTTCTCTCTCTCTCTCTCTCTCTCC

GCTATACCAAAAGTCCCCCATATCCACCCCCCCCCCCCCCCCCCCCCCCCCCCCCCCCCCTTTTTTGCCCCGGGT

TTCCTCTTCTTCCTCTTCCTCCTCCTCTTCCTCCTCTTCCTCCTCTTCCTCCTCTTCCTCTTCTTCCTTCTTCTC

TCTCTCTCTCTCTCTCTCCCTCCCTCTCCCTCCCTTCCTTCCCTTCCCTCCCCTCCCCCTCTCCTCTCCCTCTCT

GCACACACACACACACACACCTCCTACACACACACCCACATCCTACACACACCCATACACATCCTGCCCACCCAC

TGCCCTGACCTCTCCCGGCCATCCCCCTTTATCCCCTCTAGTCCACTCCCCCTCACCCCAGTCCACGTAGGCACA

CCACCTCCATCCCAACACCACCAGCCCCACTGTCAGCCCTGCCACCAACAGCACCACCAATATTACAACCAGCAC

TCTCCCTCTCCCTCTCCCTCCCTCCTCTCTGTCCCCTCTTCACCCCCTGCCTCTTTTCTTTCTCTCTTTGAGACA

GTGCTAGTCTCCTACTCTCCCTCCTCCCTCCCCCCCCTTTTCCCCCCCTCCCTCCCCTCCTCTCCCCCCCTCTCC

TGCTGGCCCAAGCCTGCCCCATCGCAGCCACCCTGCTTCTCGCCAGACCCCCCCCCCCCCCCCAAACGCCCCTGC

GACCACCACACCCCCGATCACTCACGCCCTCCGGCTCCCCCCCTCTCTCTCCTTCTCTCCCTTCCTCTCTCCCTC

TGCTCTTACTCTTACTCCTCTTGCTCTCCTCCCTCCCCTCACTCCCTTCCTCTCTCTCCCTCTTCCTCTCTAACC

ATCCAAACACACCCCCCTCCCCTCACCCCTCTCCCTGCTCACCAACGCAACCAGTCCCACTTCCTGGCTCTGGCA

CTCCGACCGAAGCCACCCGGGGCGGCCCCGCCGACCCCGAACCCGCCCCCGAGGCCCACCGACTCCAGCCGACGA

CCTTCTATTCCCCTTCTATTACGCCCCTGTGCCCCTCCCCCACCCCCCCCCCCCCCCCCCCCCTCCCCTCCCCCT

TAACAGCCCGGGCCCCGGCCCGAGCCTCTTCCCGTGGGCCCCCCTCCCTGTATCCCGGCCCGTGATCCCCGCGCC

AGCCTCCTGCCTCCAGTCCCTCCTTCTCCCTCCCTCTTCCCTCCCTCCCTCCGTTCCTCCTTCTTGCTCTCTCTC

AGTCCTCTCTCTTCCTTCCTTCCTTCCTTCCTTCCTTCCTTCCTTCCTTCCTTCCTTCCTTCCTTCCTCCCCTCC

CCTCCTTCCCTTCTTTCTCTCCCTTCTTGTCTCTTGCCCTCCCCTCCTTCCCTCCCTTCCTTCCTTCAATTCTAC

ATGCTATCCCGAAAGTTCCCTATACCCTCCCCCTGCCCTGCTCCCCTACCCACCCACTCCCTCTTCTTGGCCCTG

CCCCCTCTACCTCTCTCCCTCTCTCCCTTTCCCCATCTCCCCATCACTCCCTCCTCCCTCTCCCTCTCTTCTTCC

CCCAAAAGTCCCCCACACTCTTCCCCACTCACTCTCCCACCCATCCACTCCCACTTCTTGGCCCTGGCTTTCCCT

TCCCCCCCCCCCTCCTCCTCTTTCTCCTCTCCCCCCCTATTCCCTCCCCTCCCTTCCCCCCCTTCCCCTCCTCTC

CACACACACACACACACACACACACGCGCGCTCACACACACACACACACACACACACACACACACACACAACACC

GGCGGGGGACGGGCCCCCGCCACGCACCCCCGACCCCGAGAGACGCGCACCCGCGGCCGCTCCCGTCCCGTTCCG

TCACCTGTTCGACCCAAACATTCCGGCGACCCGGAGCGCCCGGCGCCCGCCACCCGCTCGCTCCCGGCTCCCTCT

AGCCCCCGCGCACGCCGCCGCGCTCCGCCCCGCCACCGAGTCCCCGCCGGGCGAGAGCGGCTCCACATCCGGGTA

CTTTTCTTCCCTCTTCTCCCCCCCCCCCCCAACCTCTTCTCCCCTCTTTCCTTCTTTGTCTTCCTCCTTTCTCTC

CTCCCTGTCCTTCTTCTCACCCTTCACTTCTGCCCTGACATTCATCTGACCCTCCCTTACCACCACCACCCACCC

CCCATTCCAGGTCTCCCTTTTACACACCCTCTCCTCCTTCTCCCCTTTCCCCCCTTCTCTTCCCTCCGCCCCTCC

TCCCTCCTTTCCTCCTTCCTTCCCTTCCTCCCTCCTTCCTACCTTCCCTTTCCTTCCCTGCTTCCCTTCTTTCTC

AGGTTACTTGTTGCCTCCCTCTCCCCCCTCCCCCCCCCCCCCCCCCCCCCCCCCCCTCCTCCCCCCCCCCCCCCC

CCCTTCCCCTTCCCCTCCCCTCCCCTTCTTCCTTCCTTCCTTCCTTCCTTCCTTCCTTCCTTCCTTCCTTCCTTC

GCTGCCCTTTTACCTTCTGGGCAGCCCCTGCACCACCCCCAGCACCACCACCCCCCCCCCACCCCCACTATAAGG

TTCTCCTCCTAAAACCCCCTGTCCCACACTCTCCCCCTGCTCACTAACCCACCCACTCCCGCTTCCCTGTCCTGA

ACTCTGGGCCCAAACCTAACGCCCCCCCCCCCCCCCCCCCCCCCCCCCCCCCCCCCCCCCCCCCCCTGCGAGATT

CCACTTCCCTCCCCCTCCCCCACAGCTGCCGCCAAGGAATACTCCCAAGCCCCATGCATCTCCAAAGACCCATCT

CTTCCCCCCTCCCCCTGCTCCCCAACCCACCCACTCCCACTTCCTGGCCCTGCATCTCCCTATACTGGGGCCTAG

TCCTCCTCCTCCTCCTCCTGCTCCTGCTCCTCCTGCTCTTGCTCCTGTTCCTGTTCCTCCTCCTCCTCCTGCTCC

TCCTCCTCCTCCTCCTCCTCTTCTCTTCTTCTTACTCTTCTTCCTCCTCCTCCTCCTCTTCCTCCTCCCCCTCCT

CTCCTTCTCCTCCTTCTCCTCCTTCTCCTCCTTCTCCTTCTCCTTCTTCTCCTTCTTCTCCTTCTTCTCCTTCTC

ACCACCATCACCACCATCACCACCATCACCACCATGACATCATCACCACCACCACCACCACCATCACCACCAACA

TCAAGCCCTTTGTACACCCTAAGCCTCCGCCTCCTCTTCCTCCATCCGCCCCGTCTCTCCCCCTTGAACCTCCTC

TTCCTTCCTTCCTTCCTTCCTTCCTTCCTTCCTTCCTTCCTTCCTTCCTTCCTTCCCTCCTTCCTTCTCTCTCTC

ACCACACACACACACCACACACACACACACACCCAACACACACACACACCCCACACACGCACCCACCTCCTCCAC

TCCTGGTTTCCCCTCCGAAAACCCCCTCCCCCCTCCCCCCGCTCCCCCACCCCCCCCCCCCTCCTTCCTGGCCCT

TCCTTCCTCCCTCTTTCTTTTCTTCCCCTTTCCCTTTCTGTTCTTCCCTCCCTCCCTCCCTCCCTTCCTCTCCTC

CTCTTTGTCCTCTCCTCTCCTCCCTCTTACTCTTTTCTCTAGCCTCCCCCCCTTCTACCTTCTCTCTTTCTCCCC

CATCACAGCTGTCCCCTCCCCGGCTCCCACCCTGTCAGGGTGCCCCCCCCCCTCCAACTCAGCTGCCGGAACCAT

TCCCCCTCTCTCCACATTCCCTTCCCCTACTCCACGTGCCCATGGCCAGCCTCTACTTCTCTACTCTCCCTCTCT

TCTCTCTCTCTCTCTCTCTCTCTCTCTCTCTCTATCTCTCTGTTTCTCTCTCTCCCCCCCCCTCTCTCTCTCCCT

CCTCCTGCTCCTGCTCCTCCTGCTCCTGCTCCTCCTGCTCCTTCTGCTCCTGCTCCTCCTGCTCCTGCTCCTCCT

CTCTCTCTCTCTCTCACACACACACACACACACACACTCACTCTCTCTCTCTCTCACACACACACACACACACAC

CTCCTCATGTCCCTTCCCACTCCAGTCCCCTTATGCCCCGCCCCTTGTGCCATCTAGACTACTCCCCCCCTGCAG

CCCTCACACTGTCCCTCCTCCCTCCCCTTCGCCTCTGAGAGGGCAGAGCCCCCACCCCAACCCTGGACATCCCCT

CTCTCCTCTCTTCTCCTCCTTTTCTCCTCCTCCTCCTCCTCCCCCTCCTTCTTCTCTCTCTCTCTCTCTCTCTCT

CTCAGCTCCACCCCTCTGCCCAGACCCCTGGCTCAGCTCCGCCCCTCTGTCCAGACCCCTGGCTCAGCTCCACCC

CTCCCTCCCTCCCTCTCTCTCTTCCTCTCTCTCTCTCTCTCTCTCTCTTACTCACACACACACACACACACACAC

CGCTCCTCCCTGTGCCCTTCCTGTGCTCCTCCCTGCACTCCTCCCTGTGCTCCTTCCTGCGCTCCTCCCTGCACT

CATCCACCCCACAGCTCCACATCCTACCCCTCCTCCCCACCCCACCTGACCTCTAAACTCCCTGGGGCTTCCTGT

TCTCCTCCCATACCTGATTCTGCCCCTTTTCCTCCTCCCCCTCCTCTCTTCCTTCCCAATCCCTCCCCCCCTCTC

TCCCCCCTTCCTCCTTCTTCTCCACCTCCTTCCCTTCCCCCTTTTCCTCTTCTCCCCTCCTCCTCCTCCTCCTTA

GAACCCCTTCACCCTACCCCCCCCCCCCCCCCCCCCCCCCACTCCCCCGTGGGGCCCCAGTAAAAAACAAAAGGC

TCTCCTCTCCTCTCCTCTCCTCTCCTCTCCTCTCCTCTCCTCTCTTCTCCTCTCTTCTCTGTCCTTCTCCCTTTC

TCTCTCTCCCTCTCTCTCTCTCTCCCTCTCTCTCTCTCTCTCTCTCCCCCCCACCCTCCCCCCCCCTCTCTCCTC

CGCCTCCGCCTCCGCCTCCGCCTCCGCCTCCGCCTCTGCCTGTGCACCCTCACACACTATGCACAAATATGGCTC

CCATCTCCCCTCCACTGACTGCTTCACATCCCACACCTCTTCCCCACCCCACCCCGTCTCCATGTGGATGCCCCC

CTCCAGCCCCCTCTCCCTGCCTTCACCTCTGCTCTCCATCCTCCCTTTGCTCTCTATCCTCCTTTCTCCTCTTAA

TCCAATGCTATCCCAAAAGTCCCCCACACACTCCTCCACCCACTCCCCAACCCAACCACTCCCACTTCTTGGCCC

TGTCTGCCCCATCTCCAGCACTGCCAGCTCACATGCCCTTCCCCCCAGGATCTCCCCAACCCCCATCCATCTCTG

TCCAATGCTATCCCAAAAGTCCCCCATACTCTCCACCCCACCACTCCCCTACCCACCCACTCCCACTTCTTAGCC

TTCCTCCTCCTCCTCTGCCTCCCAGTACCCTGCTTCCCAGGCCCTGCCCAGTTATCCTCATTCCTTCCCCCCACC

CCCCCATGCAGCCCCCACCCTGCCCTCCATCCCCACCCCAGCTCTACCCAAGAGGTCTTGCAGCACATCTTCTCA

ATCCCCTTTCCTAGTTTCCTCTCTGAAAGTCCCCCCCCCTATACCCTCCCCCTGCCCTGCTCCCCAACCCACCCC

GACCACCGCCGCCGCCGCCCGGCTCCGAGGGACCCGCCGGACCTGCCCCTTCCGCTGCGGCTCACTCTGCCTTGC

GCCTCCTGCCTCCTGCCTCCTGCCTCCTGCCTCCTGCCTCCTGCCTCCTGCCTCCTGCCTCCTGCCTCCTGCCTC

AAGTCCCCCACATGCTCCCCCACCCACTCCCCCCCCCCCCCCCTCCCCCTTTTTTGCCCTGCCTTTCCCCGTACT

CCTCTCTCACCCTGCCTCTCTCCCCCAGCCTCTCTCACCCGGCCTCTCTCACCCGGCCTCTCTCACCCGGCCTCT

AAATGCCATTCCCAAATCCCCCTATACCCTTCCCCCGCCCTGCTCCCCAACTCACCCACTCCAGCTTCCTGGCCC

CTTTTTTGTCTTCCTCCTCTCCTATCTCCTCCTTCTTCTCCCCCCTCCTCCTCCACTTCCTCTTCTTCCTCCTTC

Telomere

CCCTAACCCTAACCCTTAACCCTTAACCCTTAACCCTAACCCTGACCCCTAACCCTTAACCCTAACCCCTAACCC

CGGCCCGGCTCGGCTGCGCTCGGCTCCGCTCGGCCGCCCGCGCCCGCCGCCTCTTTGTTCACCGCCGCTGCCGCC

CAGGACTGGGGGCCCGGCCTCGCCCTCCCATGCCCGGCCTGGCCCGCCCCACCCGCTTTGCCTCCCACCAGGACT

CACTCCTCCTCTTCCACACTTGCCCTCCTCCCTACTCACCCTCCTCCCCACTCACCCTCCTCCCCACTCATCCTC

CCTCTCCTCCCAGTCCCCTGCACACACATACCTCGCCCCCCCCCCCCCCCCCCCCCCCCCCCCCACCCCCCCCCT

CTCTCTCTCTCTCTCTCTCTCTCTCTCTCTCTCTCTCTCTCTCTCTCCCCCCTCCCTCTTTCTCTCCCCCCCCCC

ACCACTCCACCAACCTTCCTATCCCAATTCCCCTCTCCCCCCTCCCCTTTGACTCTATGAGGATGCTCCTCCACC

TCGTTACGCCCTCCCCCACCAACGCCCCCGGCCCGGGGCACTCTTGCCACTTCATCCACCCCGGCCCCTCCCGTC

GCCCCTTTCTTCCTCTATTCTTCTCCCCCCCCCCCCTTTTCCTCCCATAAACCCCTCATCCCCATCGTACAACAA

TCACTGCCCGGCTACTTGGCTCCGCCGCCCGCTGCAGCCGCCCTGTCCTCGGCCGCTTCGCCGCCCGCCTCGACC

AGCAGATTTCCTTTCATTCCTCTGTCTCTCTACATGTTTGCTCCTGCCCCATCTCCCCCCCCCCCTCCCCCTCCC

CCCCTATCCTCTCCCTCCTCCCCCTGCTCACCAACCCACCCACTCCTGCTTCCTGGCCCTGGCATTCCCCTACAT

CTTCCTTCCTTCCTTCCTTCCTTCCTTCTTTCCTTCCTTCTTCACTCCTTCCTTCCCTCCCTCCCTCCTTCCCTC

GCTCCTCCTGCTCGGGCTCCTCCTGCTCCTCCTGCTCCTCCTGCCCCTGCTCCTCCTGCTCCTCCTGCTCCTCCT

CACCTTCTCCACTCATCCTTCCTTCCCAACCCTAAAGTCAACCACCCACCAAGCCCCACCTCACCCTACCTGTAG

CACACTGGGTTGCATTAGCCGCCCTCCCCCCCCCCCCCCCCCCCCCCCCCCTGCCCCTAACCCTTTCCCCCTTGG

GACCACGCCTCCTGACCCACCTCTCACCTGTGACCACACCCCCGAGCTCCACCCTGCCTGTCTCGCCGAGCCAGC

TGAAACACCATGACCCAAAGCTCCACATCCCACACCTCCTTCCCACCCCACCCCATCTCCCCGTGGATGCCCCCA

CCTCTGCCTCCCTGCCTCCCTGCCTCCCTCTCTGCCTCCCTGCCTCCCTCTCTGCCTCCCTGCCTCCCTCTCTGC

CGCCCCTCCTCGCCCCGCCCCGCCTCTTGGTACCCACCCGACCTTGCGCACGCGCAGCTTGTCTTCCCCCCCGGC

CGCGTGACGTGAGGCCCGCCCGCGCGCCCGCCCGGGATCCCCAGCCGCCGCCGCGCCCGCCCGCCCGGGGCCCCC

CCCTCATCTCCCCCTCCCTCCTCTTTTCCCCCCTCCACACCTCCCTTCTTTTTGCCTCTCTCGTCAATCTGTCGA

CCTCCTGCTCCTCCTCCTCCTCCTGCTCCTGCTCCTCCTCCTCCTCCTCCTCCTGCTCCTCCTGCTCCTCCTCCT

CCCTCCCTCTCCTCACAGCCTACCATCCTGCAGCCTCCAGCCCGGTGCGTCCCTCTCCGGCCACCGTAGAAGCCA

CGGGTGGCCTGAAACTGCTTCTCCCTCCCCCCCCCCCCCCCCCCCCCCCCCCCCCCCCCCATTTTCACAACTCAA

CTCCTTCTTTTCTTCCTCCTCCTCCTTCTCTTCTTCCTCCTTCTACCCCTTCCTCTTCCTCTTCCTCTTCCTCCT

CAACCACTCCCCATCCTATACCTCCCCACTTGCCCCTGTCTCCAAAAGGATGTTCACACCCCAACCCCCACCCCA

CCCACCCCTCCAGGCCTCACCCCTCCAGGGCTCACCCTTTCAGAGCCCACCCCTCCAGGGTGCACCCCGCCAGGG

CCCACTCCTTCCCTGTCCCTTTGCCTGCCCTCTCTCTCTCCCTCCCTCTCCTCCCCTCTCCTTTCTCCTTCCCTC

CTCTAAAGGTGTTCTGCGCTCCCCACTCCCTCTCCCTCTCCCCCTCCCCCCCCCCCCCCCCCCCCCTCCCTCCCC

CTTCCTCTTCCCCTCCCTTTTCCCTTCCTCTTCCCCTTCCTTTCCCCTCCCTCTCCCTTTCCCTCTCTCTCACCA

TTCCTCCATCCGCCCCGTCTCTCCCCCTTGAACCTCCTCGTTCGACCCCGCCTCGATCCTCCCTTTATCCAGCCC

CCAATGCTATCACAAAAGTCCCCCATACCTTCCCCCACACACTCCCCTACCCACCCACTCCCACCTCTTGGCCCT
