# Supplementary material for: Heterogeneous nuclear ribonucleoprotein E1 binds polycytosine DNA and monitors genome integrity
Source: Life Sci Alliance. 2021 Jul 16;4(9):e202000995. doi: 10.26508/lsa.202000995 (PMC8321654; doi:10.26508/lsa.202000995)
Supplement: Supplementary file 2 [file LSA-2020-00995_TableS2.docx]

**Supplementary materials**

**Table S2. List of (deoxyribo and oxyribo) oligonucleotides used in the experiments.**

1. BAT RNA

5’-r(AAUUGCUACCCAAUGCCUGGCCCAAGGGCAUUCCCAAAGCUUA) -3’

1. TEL DNA C

5’-TAACCCTAACCCTAACCCTAACCCTAACCCTAA-3’

1. TEL DNA G

5’-TTAGGGTTAGGGTTAGGGTTAGGGTTAGGGTTA-3’

1. c-MYC C

5’-CCTTCCCCACCCTCCCCACCCTCCCCA-3’

1. c-Myc G

5’-TGGGGACCCTGGGGAGGGTGGGGAAGG-3’

1. BCL2 C

5’CAGCCCCGCTCCCGCCCCCTTCCTCCCGCGCCCGCCCCT-3’

1. HIF-1α C

5’CGCGCTCCCGCCCCCTCTCCCCTCCCCGCGCGCCCGAGCGCGCCTCCTCCCTTCCCGCCCCCTG-3’

1. EGFR-272 C

5’-CCCAGCACTGCCCCTCTGGACCCGGTCCCC-3’

1. PDGF A C

5’-CCGCCGCCGCCGCGCCCCTCCCCCGCCCCCGCCCCCGCCCCCCCCCCCCCG

CCTCCCCGG-3’

1. VEGF C

5’-CTCCGCCCCGCCGGGACCCCGCCCCCGGCCCGCCCC-3’

1. OK1

5’-CCCTAACCCTAACCCTAACCCTAAGACTCAGTCGAG-3’

1. OK2

5’-CTCGACTGAGTCTTAGGGTTAGGGACGTCGATACGTAGCTGATC-3’

1. OK4

5’-GATCAGCTACGTATCGACGTG-3’

1. ODN-1

5’-TTTTTGTGTTTTTTTTTGCAATTTTT-3’

1. ODN-2

5’-CGTCCGTGTCCTGCGTCCGCAATCCGA-3’

1. ODN-3

5’-CGCCCGTGCCCTGCGCCCGCAGGGCGA-3’

1. hras-2^Y^

5’-ACCGCGCGCCCCCGCCCCCGCCCCGCCCCGGCCTCG-3’

1. hras-1^Y^

5’-CGCCCGTGCCCTGCGCCCGCAACCCGA-3’

1. myc-chipTQ F (for PCR)

5’-CTACGGAGGAGCAGCAGAGAA-3’

1. myc-chipTQ R (for PCR)

5’-GCCTCTCGCTGGAATTACT-3’

1. EGFR F

5’-TTGGCTCGACCTGGACATAG-3’

1. EGFR R

5’-TTAATTTCCGAGAGGGGCGTT-3’

1. H-Ras2Y F

5’-GGCTCCTGACAGACGGG-3’

1. hRAS2Y R

5’-GCATGGGCTCCGTCC-3’
